# Solid Medication Intake in Hospitalised Patients With Dysphagia: A Challenge for Speech and Language Pathologists?

**DOI:** 10.1111/1460-6984.70073

**Published:** 2025-07-02

**Authors:** Michaela Trapl‐Grundschober, Lea Schneider, Steffen Schulz, Simon Sollereder, Yvonne Teuschl, Walter Struhal, Jürgen Osterbrink

**Affiliations:** ^1^ Karl Landsteiner University of Health Sciences Krems Austria; ^2^ Division of Neurology University Hospital Tulln Tulln Austria; ^3^ Paracelsus Medical University Doctor of Philosophy (PhD) Program in the Institute for Nursing Science and Practice Salzburg Austria; ^4^ Brothers of St. John of God Hospital Eisenstadt GmbH Eisenstadt Austria; ^5^ EUFH Campus Rostock, University of Applied Sciences Rostock Germany; ^6^ VASCage Centre on Clinical Stroke Research, Division for Rehabilitation & Recovery Innsbruck Austria; ^7^ Department for Clinical Neurosciences and Preventive Medicine University for Continuing Education Krems Krems Austria

**Keywords:** dysphagia, dysphagia evaluation, medication intake, pill dysphagia, speech and language pathologist, stroke

## Abstract

**Purpose:**

Speech and language pathologists (SLP) are frequently consulted for guidance on the management of oral solid medications. However, the extent to which SLPs consider solid medication intake during swallowing examination remains unclear. The present study endeavoured to find out whether and how SLPs assess patients’ ability to swallow solid dosage forms (SDF) in clinical settings, with a specific focus on stroke units.

**Methods:**

An online cross‐sectional survey was conducted among SLPs in German‐speaking countries. The questionnaire was targeted at SLPs working in hospitals and distributed through both direct outreach and passive dissemination on social networks.

**Results:**

Of 200 returned questionnaires, 147 were eligible for inclusion. Of these, 108 (73.5%) evaluated the swallowing ability of solid oral medications and were further analysed. During clinical swallowing examination, the patient's own medication is most frequently used (63.9%). 92.6% of the 108 SLPs stated that they regularly offer guidance on altering SDFs. SLPs' decision to recommend modified SDFs or pause them is influenced by oral cavity retention of SDFs, pharyngeal phase disorders and SDF intake‐related coughing. Additionally, SLPs employ textures that were determined to be safe for accompanying boluses, with fruit puree and water being the most preferred types. Responses of SLPs working on stroke units did not differ significantly from those working on other wards.

**Conclusion:**

SLPs commonly evaluate the swallowing ability of SDFs. Typically, they utilise the patient's own medications for testing purposes and previously trialled consistencies as accompanying boluses. Specific evaluation and educational programs are needed to enhance the management quality of oral SDFs.

**Trial Registration**: ClinicalTrials.gov identifier: Registration ID: NCT05173051/Protocol ID: 11TS003721

**WHAT THIS PAPER ADDS:**

*What is already known on the subject*

- Speech Language Pathologists are crucial in assessing and managing dysphagia. Recent studies and guidelines suggest that it is important for SLPs to evaluate the ability to swallow solid medications. However, there is limited research on whether and how SLPs assess and manage solid dosage forms in patients with dysphagia, especially those with post‐stroke dysphagia. Although a few studies have used instrumental assessments like FEES to evaluate pill swallowing, there is a lack of standardised methods.

*What this paper adds to existing knowledge*

- The findings indicate that almost three‐quarters of the surveyed SLPs do evaluate SDFs, with fruit puree and water being the most preferred accompanying boluses. Patients' own medications are more frequently used in clinical swallowing examinations (CSE), whilst placebos are preferred in instrumental assessments. A large majority of SLPs are involved in deciding whether solid medications should be modified for patients with dysphagia.

*What are the potential or actual clinical implications of this work?*

- The research highlights the need for standardised protocols for the evaluation of swallowing SDFs as part of both clinical and instrumental assessments. Additionally, there is a critical need for the establishment of professional and regulatory guidelines to ensure consistent and evidence‐based practices among SLPs, potentially improving patient care and medication administration.

## 1 Introduction

Speech and language pathologists (SLPs) play a pivotal role in assessing and diagnosing swallowing disorders, as they possess specialised knowledge and skills to identify, characterise and manage dysphagia‐related issues. They apply various diagnostic tools, including screening methods and the more detailed and comprehensive clinical swallowing evaluation (CSE) (Speyer et al. 2022). Advanced instrumental techniques such as video fluoroscopic swallowing study (VFSS) or fibreoptic endoscopic evaluation of swallowing (FEES) allow for real‐time visualisation of the swallowing process and are considered gold standards. SLPs are responsible for determining the appropriateness of oral intake by testing bolus trials of different amounts and textures during the CSE, assisting in the recommendation of the safest consistencies for oral diet (Garand et al. 2020; Speyer et al. 2022).

Although these assessments routinely address food and liquid textures, the systematic evaluation of solid medications remains largely absent. Although guidelines recommend evaluating the swallowing ability of solid dosage forms (SDFs) and identifying optimal formulations, no standardised approach has been established within the CSE or other screening procedures (Dziewas et al. 2021; Powers et al. 2019; National Institute for Health and Care Excellence (NICE) 2019). In everyday clinical practice, however, SLPs are frequently confronted with the question of whether dysphagic patients can safely swallow their medications (Kelly and Wright 2010; Masilamoney and Dowse 2018). Despite working closely with physicians, pharmacists and nurses to determine safe administration strategies, SLPs still operate without a standardised framework guiding their role in medication recommendations. In addition, the referral process for medication‐related swallowing assessments is not clearly defined, and coordination between SLPs and other healthcare professionals varies widely between institutions (Robinson et al. 2022; Manrique et al. 2014; O'Hara 2015).

As instrumental assessments like FEES and VFSS provided greater insight into swallowing physiology, SLPs have become increasingly involved in medication‐related decisions, particularly in determining safe administration methods. This has led to instrumental assessment tools being proposed as the preferred method for evaluating the swallowing ability of SDFs, with some authors advocating for the standardised use of placebos in every examination (Wirth and Dziewas 2019; Brady 2015). An exploratory literature search revealed that only four studies have delved into the use of medication intake with FEES (Schiele et al. 2015; Carnaby‐Mann and Crary 2005; Buhmann et al. 2019; Labeit et al. 2022). Schiele and colleagues investigated 52 stroke patients in a subacute stage. In their study, 40.4%–43.5% of patients experienced severe difficulties swallowing SDFs. They discovered the risk of aspiration was significantly higher when tablets were ingested with both water and pudding, compared to swallowing these consistencies separately. Overall, pill/capsule swallowing with thickened water was safer than swallowing with milk. Compared with FEES, neither patients’ self‐assessment nor conventional clinical swallow tests were able to detect difficulties with pill swallowing (Schiele et al. 2015). Another study was performed by Carnaby et al. on a cohort of 36 patients who presented with dysphagia resulting from various conditions. They evaluated the swallowing physiology and safety, comparing a conventional tablet and a new method of tablet transportation. They discovered that patients with dysphagia demonstrated significantly longer total swallow durations, a higher number of swallows per tablet and the need for fluid to assist in the clearance of the conventional tablet (Carnaby‐Mann and Crary 2005). Buhmann conducted a study involving 118 patients with Parkinson's disease and 32 controls using FEES to investigate their ability to swallow four placebo tablets. They found that 28% of patients with Parkinson's disease had significant difficulty swallowing pills, with capsules being the easiest to swallow and oval tablets being the most challenging (Buhmann et al. 2019). This phenomenon was also confirmed in a study by Labeit and colleagues, who revealed that swallowing capsules was more efficient than swallowing tablets (Labeit et al. 2022). They additionally indicated that the prevalence rate of dysphagia for medication among patients diagnosed with Parkinson's disease is approximately 70%. It is worth noting that Yamamoto et al. (2014) conducted the only VFSS study on the behavioural performances of tablet swallowing, exclusively on 14 healthy young participants. The group demonstrated that four different types of tablets (small or large, with or without a surface coating) affected the swallowing function in terms of total number of swallows, electromyographic burst patterns and location of remaining tablets (Yamamoto et al. 2014).

The scarcity of empirical evidence regarding the swallowability of whole and modified SDFs through objective instrumental procedures has led to a reliance on experience‐based rather than evidence‐based approaches (Blaszczyk et al. 2023; Daibes et al. 2023). Moreover, the optimal design of pills/capsules (e.g., in terms of their size, shape and surface) remains insufficiently investigated in dysphagic patients and was only shown in healthy individuals, who generally preferred medium‐sized pills/capsules with a smooth surface (Fields et al. 2015). As a result, inappropriate modification of oral medication, such as crushing, opening capsules or breaking tablets, is often observed and can lead to medication errors (Masilamoney and Dowse 2018; Wirth and Dziewas 2019; Wright et al. 2020). This “off‐label” preparation may distort pharmacodynamics and may harm the gastrointestinal tract (Logrippo et al. 2017). To address this, some authors suggest involving SLPs if swallowing difficulties occur and to work together with pharmacists to tailor drug therapy to the swallowing requirements of patients (O'Hara 2015; Wright et al. 2020).

Although no formalised protocol currently exists for the clinical assessment of SDFs, growing awareness of this issue is reflected in white papers and professional guidelines that acknowledge the relevance of pill‐swallowing difficulties, yet without providing concrete assessment proposals (Wright et al. 2020; Speyer et al. 2022; Dziewas et al. 2021). In this context, two validated self‐reported questionnaires were published in the last 7 years (Arnet et al. 2020; Messerli et al. 2017; Nativ‐Zeltzer et al. 2019). The SWAllowing difficulties with MEDication intake and COping strategies (SWAMECO) questionnaire was published by Swiss pharmacists in 2017 and contains 30 items divided into five sections focusing on issues related to swallowing difficulties with medication intake (complaints, intensity, localisation, coping strategies and adherence). They conducted both face and content validation with a group of eleven experts and nine systemic sclerosis patients, respectively, but without instrumental testing (Messerli et al. 2017). In 2019, a validated 5‐item patient‐reported outcome measure for detecting pill dysphagia was published by Nativ‐Zeltzer and colleagues (“Pill‐5”). However, among 190 included patients, only ten individuals were affected by neurogenic dysphagia, whilst the remaining patients suffered from oesophageal dysphagia diagnoses (Nativ‐Zeltzer et al. 2019).

Notably, two broader dysphagia assessments, the Dysphagia Handicap Index and the Munich Dysphagia Test‐Parkinson's Disease, have incorporated questions on pill‐swallowing difficulties, illustrating initial steps towards embedding this aspect into comprehensive evaluation tools, although specific, standalone protocols for SDF assessment remain lacking (Silbergleit et al. 2012; Simons et al. 2014).

There is limited data available to support SLP's decision on the appropriate bolus size and type with which SDFs can be administered. According to Schiele and colleagues’ research, the risk of penetration and aspiration of an accompanying bolus is significantly higher when it is administered with SDFs than when it is swallowed alone (Schiele et al. 2015). In clinical practice, thickened water, apple sauce or milk products are frequently utilised for this purpose (Manrique et al. 2014). Some research has shown that applesauce and orange juice can greatly alter the absorption and bioavailability of some medicines (Yin et al. 2011; Petric et al. 2020; Iftikhar et al. 2024). Furthermore, recent research suggests that thickeners may influence drug release; however, current evidence indicates that this effect is medication‐specific and not yet fully understood (Malouh et al. 2025). Thickeners are commonly used in hospitals and geriatric wards to reduce the flow rate of fluids and prevent aspiration in dysphagic patients. Due to the limited research available, the potential impact of thickened consistencies on drug efficacy remains unclear and requires further investigation (Royal College of Speech and Language Therapists 2024; Atkin et al. 2024; Ilgaz et al. 2022; Malouh et al. 2025).

On stroke units, initial diagnostic tests for dysphagia often involve non‐instrumental assessments, which include screening methods and the CSE. These tests are typically the first line of assessment for patients suspected of suffering from dysphagia and should be done as soon as possible after admission (Dziewas et al. 2021). SLPs are expected to recommend an appropriate diet as well as the mode of SDF delivery. Some studies and guidelines suggest that in patients kept nil per os because of severe dysphagia, drugs should not be given orally until their oral intake has been specifically tested and approved with an instrumental method, such as FEES (Schiele et al. 2015; Leder and Lerner 2013; National Institute for Health and Care Excellence (NICE) 2019). It is currently unclear how SLPs typically evaluate the ability of patients with post‐stroke dysphagia to swallow SDFs, as well as the recommendations provided by SLPs following such assessments. Given the paucity of research on this topic, it remains essential to examine the current state of clinical practice in this area. Understanding the strategies employed by SLPs in the evaluation of SDF administration can provide valuable insights into the optimal management of patients with dysphagia.

The current study explores the comprehensive assessment and diagnosis of swallowing disorders by SLPs, with a specific focus on the challenges and considerations associated with SDFs. This study has two aims: (1) To investigate (1a) whether and (1b) how SLPs in German‐speaking countries assess the swallowing ability of SDFs as part of dysphagia diagnostics in a clinical setting, and whether there are (1c) differences in stroke units; and (2) To ascertain which parameters are used by SLPs to make recommendations regarding the management of SDFs in patients with dysphagia.

## 2 Materials and Methods

### 2.1 Aim

The current investigation constitutes a segment of a broader project that aims to scrutinise the oral intake of SDF amongst patients with acute stroke and dysphagia. It includes two web‐based surveys and a clinical observational study on solid medication intake and management. The primary objective of the current paper is to comprehensively examine and analyse the methods used by SLPs in diagnosing and managing SDFs.

### 2.2 Design

A quantitative cross‐sectional study was conducted using a self‐administered web survey among SLPs working in clinical settings in German‐speaking countries.

### 2.3 Sampling Method

A convenience sampling technique was used, which entailed both active and passive methods to reach clinically employed SLPs who also specialise in treating patients with post‐stroke dysphagia. The sampling method was designed to ensure quick and efficient data acquisition from the target population. In an effort to connect with a wide range of clinically active SLPs working on stroke units, some hospitals were contacted directly. However, this approach did not have an all‐encompassing reach, as some stroke units were not considered.

### 2.4 Data Collection

A structured online survey consisting of 27 questions was developed with the assistance of the survey tool Qualtrics (Qualtrics 2005). All questions of the questionnaire can be found in Supporting Information S1 and S2. Six superordinate topics encompassed (1) diagnosis of SDFs during both instrumental and non‐instrumental assessments, (2) methods used for evaluation of SDFs, (3) determination of accompanying boluses when taking whole or crushed tablets, (4) decision‐making processes based on simulated patient cases, (5) the influence of pathomechanisms on medication management decisions and (6) self‐assessed knowledge on SDF management. Topic 4 has been excluded from the analysis as it comprises a qualitative evaluation intended for separate publication. The questionnaire featured Likert items and multi‐categorical response formats. Where needed, the questionnaire employed a mix of closed and open‐ended questions, along with ‘other’ and ‘comment’ options, to guarantee that no vital additional information was missed.

A pretest of the questionnaire was performed by three healthcare professionals and adapted according to their suggestions. The final version of the questionnaire was distributed between February and March 2022. Initially, the survey was shared on various social media groups to gather responses from SLPs in Germany, Austria, Switzerland, Liechtenstein and South Tyrol. The groups varied in size from 800 to 2500 members and were categorised as either professional (SLP) or dysphagia‐specific. Additionally, the survey link was actively emailed to SLPs working in Austrian hospitals to augment participation rates. Considering the low response rate from Switzerland, individual SLPs from the country were personally invited via email to complete the questionnaire and forward it to their respective professional contacts. Due to the substantial response rate from German SLPs in the Facebook Dysphagia expert group, which at the time had a membership exceeding 2500 individuals, a separate invitation targeted SLPs at German clinics was not considered necessary. The survey remained accessible for three weeks, open to all who desired to participate, and was subsequently closed on 4 March 2022.

### 2.5 Data Analysis

All patients who were employed in a hospital during the survey and provided their consent for their data to be used anonymously were included in the study. The survey, therefore, started with two specific questions to filter the responses. Questionnaires containing a significant number of unanswered questions, that is, exceeding 50% of the total number of questions, have been excluded from the analysis. Missing responses were presented in the diagrams as ‛others’ or ‛missing’. All results were presented in percentages and absolute numbers. All data were exported from Qualtrics to IBM SPSS Statistics version 29. Descriptive diagrams were generated with the Qualtrics questionnaire software, but also via Excel for Mac (Version 16.81) or SPSS. The subgroup of 65 SLPs who reported evaluating SDFs and working on a stroke unit was compared and analysed with the rest of the population (*n* = 43) using descriptive statistics and the Chi‐square test. Furthermore, the Chi‐square test was also used to analyse the use of placebos versus patients’ own medication in CSE versus instrumental assessment. The alpha level was set at 0.05. Cramer's V was calculated as a measure of effect size, relying on the standards of Cohen 1988 (0.1 to <0.3: small; 0.3 to <0.5: medium; 0.5: large effect) (Cohen 1988).

### 2.6 Ethical Approval and Data Protection

The study was approved by an independent ethics committee (Approval available upon request). Data were anonymised and securely stored in accordance with institutional and national data protection guidelines.

## 3 Results

**FIGURE 1:**
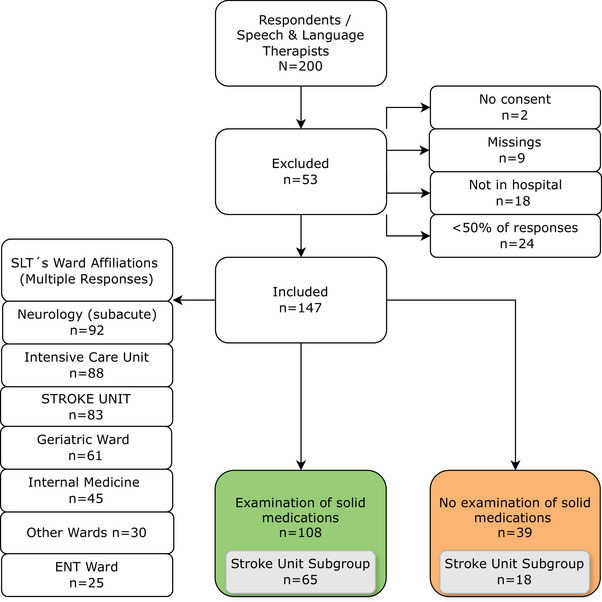
Flowchart of participant inclusion and their affiliations (multiple responses possible).

A total of 200 SLPs participated in the survey. Among them, 53 were excluded from analysis, mostly because they did not work in a hospital or because less than 50% of questions were completed (Figure 1). Data of the remaining 147 respondents were entered for further analysis. The survey sample contained individuals from three German‐speaking countries: 82 from Germany, 60 from Austria and 5 from Switzerland. No responses were received from Liechtenstein and South Tyrol. The vast majority of SLPs (66.7%) had less than 10 years of professional experience. Respondents with 11–20 years of experience accounted for 23.1%, whilst 10.2% of SLPs reported having more than 20 years of experience. As SLPs may not exclusively be dedicated to a particular ward, the survey allowed multiple responses. Figure 1 illustrates that the majority of SLPs work predominantly in neurological wards (*n* = 92), intensive care units (*n* = 88) and stroke units (*n* = 83).

Of the 147 participants, 108 (73.5%) reported conducting swallowing assessments for SDFs, whilst 39 (26.5%) did not. Among those 108, 71 (48.3%) integrated this assessment into their routine clinical dysphagia evaluations, whereas 37 (25.2%) performed it only in exceptional cases.

The most frequently cited exceptional cases in which SDFs are tested were: Coincidence of CSE and time for medication intake (*n* = 5), at the request of nurses/ward; observed difficulties by nurses or patients (*n* = 7), in case of anomalies (medical history, various textures) (*n* = 6), during FEES (standardised, with Placebos) (*n* = 6). All answers are listed in detail in German and English in Supporting Information S3.

When asked whether patients with suspected dysphagia receive SDFs prior to the SLP consultation, 91 individuals (61.9%) admitted to this practice.

### 3.1 SLPs Who Stated Not to Assess SDFs

Among the total of 147 respondents, 39 reported that they do not assess SDFs. The most frequently cited reason for not evaluating SDFs was the lack of standardised assessment tools for this area (*n* = 23). Not a single respondent believes that SDF evaluation falls outside the scope of speech therapy. Four people mentioned that institutional policy prohibits SLPs from being responsible in this field, whilst three indicated that assessing solid textures could guide solid medication intake. Moreover, a significant majority of 74.1% (*n* = 29) recommend a method for administering SDFs, although they stated not to evaluate them specifically.

All subsequent analyses are based on the 108 SLPs who reported assessing SDFs, with the subgroup analysis of SLPs working in stroke units presented separately.

### 3.2 Type of Assessments Which Are Used for the Evaluation of SDFs (*n* = 108)

**FIGURE 2:**
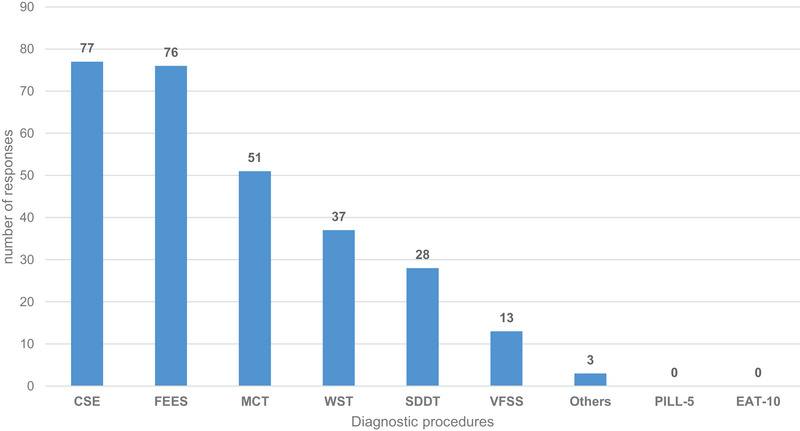
Diagnostic procedures that serve as the basis for diagnosing the swallowing ability of solid dosage forms (Multiple responses from 108 SLPs), bars represent absolute numbers. CSE indicates clinical swallow examination; FEES, fibreoptic endoscopic evaluation of swallowing; MCT, multiconsistency test; WST, water swallowing test; SDDT, Self‐designed diagnostic tool; VFSS, videofluoroscopic swallowing study.

Most of the 108 participants who reported conducting evaluations revealed using the clinical swallowing examination (*n* = 77) and FEES (*n* = 76) as the foundation for diagnosing the swallowing ability of SDFs, with some utilising both approaches (Figure 2).

**FIGURE 3:**
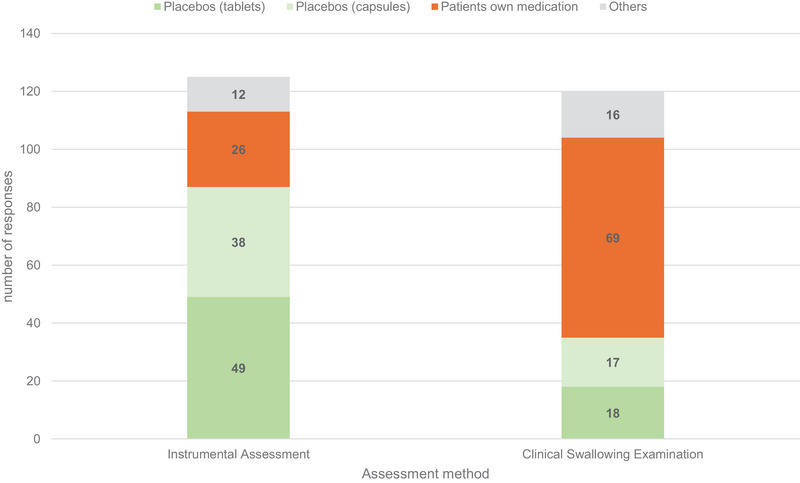
Type of assessment and type of tablets used for evaluation; multiple responses from 108 SLPs.

When queried about what kind of SDFs were used with which assessment, a substantial proportion of participants reported utilising the patient's own medications during CSE (*n* = 69); conversely, only 26 employed these medications during instrumental assessments. By contrast, 87 respondents confirmed using placebos (capsules and tablets) during instrumental assessments, whilst only 35 use them for clinical evaluation. These frequencies were significantly different χ^2^ (*df* = 3, *n* = 108) = 42.31, *p* < 0.001. The strength of this effect, measured by computing Cramer's V, can be classified as medium (*V* = 0.42), according to Cohen's (1988) guidelines (Figure 3).

Additionally, 51 (47.2%) SLPs stated that to evaluate patients’ own medication in a crushed form, whilst only 16 (14.8%) reported using crushed placebos. Since multiple responses were possible, 59 participants (54,6%) used at least one of these methods, with a subset utilising both.

### 3.3 Recommendations of Alternative Methods for Medication Administration

The findings reveal that 92.6% (*n* = 100) of those 108 SLPs who conduct assessments of SDFs reported recommending whether solid medications should be modified (e.g., crushed, divided, opened). Table 1 displays that SLPs are also involved when oral medications should be paused because of severe dysphagia, as well as when a nasogastric tube is needed. All seven SLPs who selected the ‛Others’ option have indicated that their responsibilities involve providing recommendations, as opposed to making decisions related to alternate administration.

**TABLE 1:** Recommendations on type of medication administration (*n* = 108) (multiple responses).

	Responses		
	*N* (SLPs)	%	% of SLPs
**Recommendations**			
Other dosage form	78	18.6%	72.2%
Paused	81	19.3%	75.0%
NGT	79	18.8%	73.1%
**Oral‐modified**	**100**	**23.8%**	**92.6%**
Oral‐solid	75	17.9%	69.4%
Others	7	1.7%	6.5%
Total	420	100.0%	388.9%

*Note*: The bold print shows the item with the most frequent mentions.

Abbreviation: NGT, nasogastric tube.

### 3.4 Pathomechanisms and Pathological Signs Influencing SLP´s Recommendations

The group of respondents who evaluate SDFs (*n* = 108) was asked which pathomechanisms or symptoms influence their decision to recommend modified SDFs or pause them. From the data in Table 2, it is apparent that disorders of the pharyngeal phase (*n* = 95), cough during or after SDF intake (*n* = 90) and SDFs which remain in the oral cavity (*n* = 97) have the most impact on SLP recommendations.

**TABLE 2:** Pathomechanisms and pathological signs influencing SLP's recommendations to modify or pause solid medications (*n* = 108).

	Low to very low impact	Neutral	High to very high impact	Applicable
	*n* (%)	*n* (%)	*n* (%)	*n* (%)
**Disorders of individual phases of swallowing**				
Oral preparatory phase	46 (48.9)	12 (12.8)	36 (38.1)	94 (100)
Oral phase	8 (8.0)	12 (12.0)	80 (80.0)	100 (100)
Pharyngeal phase	1 (1.0)	3 (3.0)	**95 (96.0)**	99 (100)
Esophageal phase	26 (28.3)	18 (19.6)	48 (52.2)	92 (100)
**Dysphagia and/or aspiration signs**				
Cough during or after SDF intake	2 (2.0)	6 (6.1)	**90 (91.8)**	98 (100)
SDFs remain in the oral cavity	1 (1.0)	1 (1.0)	**97 (98.0)**	99 (100)
Chewing on SDFs	17 (18.1)	13 (13.8)	64 (68.1)	94 (100)
Expectoration of SDFs	8 (8.3)	7 (7.3)	81 (84.4)	96 (100)
**Disorders of specific swallowing‐related cranial nerves**				
Facial nerve	49 (5.1)	18 (20.2)	22 (24.7)	89 (100)
Hypoglossal nerve	26 (27.1)	16 (16.7)	54 (56.3)	96 (100)
Glossopharyngeus and vagal nerves	13 (14.0)	14 (15.1)	66 (71.0)	93 (100)
Trigeminus nerve (Sensitivity)	18 (18.9)	17 (17.9)	60 (63.2)	95 (100)
Impairment of more than one cranial nerve	9 (9.8)	11 (12.0)	72 (78.3)	92 (100)

*Note*: The bold printed numbers indicate the three most frequently mentioned items.

Abbreviation: SDF, solid dosage forms.

### 3.5 Accompanying Boluses

The most widely used consistencies of accompanying boluses for SDF delivery are those that have previously been proved safe during testing (*n* = 67). Frequently used consistencies were IDDSI 4 (extremely thick) and IDDSI 0 (thin), with 33 and 30 mentions, respectively (Table 3). In a further question on the specific boluses, a majority of the participants, specifically 83, resorted to utilising fruit puree, such as apple sauce, followed by water (*n* = 71) and thickened water (*n* = 58) (Table 3).

**TABLE 3:** Choice of consistency and type of accompanying boluses for evaluation of SDFs, multiple responses (*n* = 108).

	Responses				Responses		
	*N*	%	% of cases		*N*	%	% of cases
**Consistencies**				**Bolus_type**			
Only safe tested textures	67	40.1%	62.0%	Fruit puree (e.g., apple sauce)	83	29.5%	76.9%
IDDSI 4 (extremely thick)	33	19.8%	30.6%	Water	71	25.3%	65.7%
IDDSI 0 (thin)	30	18.0%	27.8%	Thickened water	58	20.6%	53.7%
IDDSI 2 (mildly thick)	15	9.0%	13.9%	Yoghurt	27	9.6%	25.0%
IDDSI 3 (moderately thick)	14	8.4%	13.0%	Porridge	21	7.5%	19.4%
IDDSI 1 (slightly thick)	5	3.0%	4.6%	Babyfood	12	4.3%	11.1%
Others	3	1.8%	2.8%	Medication lubricant gels	6	2.1%	5.6%
				Others (jelly [2] & available food [1])	3	1.1%	2.8%
Total	167	100.0%	154.6%	Total	281	100.0%	260.2%

Abbreviations: IDDSI, International Dysphagia Diet Standardisation Initiative; SDFs, solid dosage forms.

### 3.6 Knowledge of SDF Management

When queried about SLPs’ proficiency in diagnosing and managing SDFs in patients with dysphagia, the majority of SLPs expressed that they possess average (*n* = 32) to good (*n* = 46) levels of expertise on a 5‐point Likert item. Furthermore, 72 (66.6%) individuals expressed a desire for more training on this topic.

### 3.7 Stroke Unit Subgroup

**FIGURE 4:**
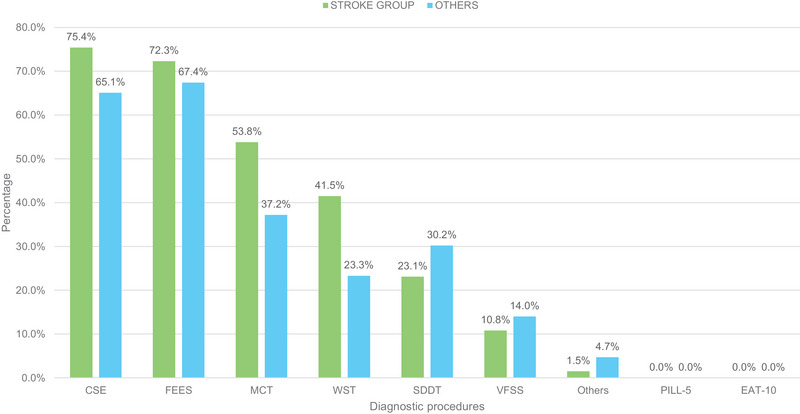
Comparison of diagnostic procedures used by the stroke group (*n* = 65/100%) versus others (*n* = 43/100%) for the evaluation of the swallowability of solid dosage forms (SDFs). CSE indicates clinical swallow examination; FEES, fibreoptic endoscopic evaluation of swallowing; MCT, multiconsistency test; SDDT, self‐designed diagnostic tool; VFSS, videofluoroscopic swallowing study; WST, water swallowing test.

A comparative analysis was conducted between SLPs working primarily in stroke units (*n* = 65) and those working exclusively in other wards (*n* = 43) (Figure 1). A higher percentage of SLPs working in stroke units assess SDFs (78.3%) compared to those working in other wards (67.2%); however, this difference was not statistically significant (*n* = 147; χ^2^ = 1.76; p = 0.18). As a foundation for diagnosing SDFs, the most mentioned procedures of the stroke population are CSE (75.4%) and FEES (72.3%). Both multiconsistency (MCT) and water swallowing tests (WST) assume greater importance, particularly in the stroke area, when compared to the remaining group working on other wards (Figure 4). However, this difference was not statistically significant (*n* = 88; χ^2^ = 0.04; *p* = 0.84). There was no substantial difference between the two groups in terms of using the patient's own medication for evaluation. 61.5% of the stroke unit group, compared to 67.4% of the remaining group, indicated using the patient's own medication for evaluation, which was like the total population (63.9%). Both groups revealed a similar preference for accompanying bolus types: Fruit puree (e.g. apple sauce) (Stroke group: 72.3% vs. 83.7%), followed by water (Stroke group: 70.8% vs. 58.1%) and thickened water (Stroke group: 55.4% vs. 51.2%) are the most frequent. In contrast, 70.8% of SLPs working on stroke units only use previously tested consistencies as accompanying boluses, compared to 48.8% of the remaining group. After comparative analyses of all items, no further statistically significant differences were found between SLPs working in stroke units and those working in other wards.

## 4 Discussion

This research is an initial attempt to investigate the extent to which SLPs consider the assessment of SDF. Findings from German‐speaking countries reveal considerable uncertainty and inconsistency in current practice, primarily due to the absence of standardised assessment tools and guidelines. Although focusing on German‐speaking countries, the issue is internationally relevant across healthcare systems. The underlying challenges—such as unclear professional responsibilities, limited interdisciplinary collaboration and lack of pharmacological training—may also affect other healthcare systems and require further investigation in both international and national contexts. Dysphagia management holds high priority in several countries—for example, in the United States and the United Kingdom—where SLPs are actively involved in patient care. However, medication intake assessment is not explicitly addressed in professional guidelines. Although the American Speech‐Language‐Hearing Association (ASHA) and the Royal College of Speech and Language Therapists (RCSLT) define SLP competencies in dysphagia management, neither provides specific guidance on evaluating medication intake, leaving SLP involvement dependent on institutional policies and local regulations. As a result, practices vary widely and remain unpublished, preventing the development of standardised protocols (American Speech‐Language‐Hearing Association 1997–2025; Royal College of Speech & Language Therapists 2025). This underlines the need for international consensus on standardised SDF assessment protocols to guide clinical decision‐making in dysphagia management across diverse healthcare systems.

The results of this survey demonstrate that a significant number of SLPs incorporate the assessment of solid oral medications into their clinical and instrumental swallowing examination. Although the regular assessment of SDFs as part of the CSE is common, there is a lack of data, guidelines and recommendations to support it. Besides CSE, FEES is the second most mentioned assessment method for diagnosing the swallowability of SDFs. This finding is in accordance with recent studies that explicitly advocate the testing of SDFs during FEES investigation (Wirth and Dziewas 2019; Brady 2015; Dziewas et al. 2024).

Interestingly, a substantial proportion of SLPs who denied evaluating SDFs still recommend methods for administering SDFs. Some of these respondents explained that they used the evaluation of solid textures as a method to estimate the risk of swallowing SDF. However, there is no data to support this approach.

One unanticipated result was that a significant number of SLPs use the patients’ own medication to evaluate SDFs during CSE. This practice may be attributed to SLPs frequently visiting patients during mealtime when medication is also administered (Speyer et al. 2022). Another explanation might be that nurses often seek guidance from SLPs on administering tablets to dysphagic patients due to a lack of pharmacological knowledge, available standards and other uncertainties (Kelly and Wright 2010). Previous studies have also highlighted that nurses frequently rely on both SLPs and pharmacists for advice on medication administration in patients with swallowing difficulties (Sefidani Forough et al. 2020; Mc Gillicuddy et al. 2017). This is supported by the results of a recently published survey study of nurses conducted as part of this project. In this study, 42.1% of nurses working in stroke units reported waiting for an expert before administering oral intake (Trapl‐Grundschober et al. 2025).

Although SLPs are responsible for the diagnostic aspect of the swallowability of SDFs, monitoring medication intake is a key role of nurses (Hanson and Haddad 2024). This represents an interface between the professional groups that has not yet been considered in the literature or in professional and regulatory guidelines. From a medicolegal perspective, this overlap creates uncertainty, as the use of patients’ own medications for assessment purposes by SLPs is not explicitly regulated, although the evaluation of swallowing safety is part of their legal mandate. This legal ambiguity underscores the need to adapt regulatory frameworks to current clinical practice.

A majority of SLPs reported providing advice on medication modifications. Previous studies have also identified SLPs as nurses’ preferred source of guidance for medication administration in patients with dysphagia (Kelly and Wright, 2010). This contrasts with SLPs receiving little or no formal training in pharmacology. Additionally, several studies emphasise the importance of carefully considering whether medication modifications are safe, as improper alterations may compromise drug efficacy or increase the risk of adverse effects (Masilamoney and Dowse 2018; Daibes et al. 2023). It remains unclear to what extent SLPs interact with pharmacists and physicians regarding the modification of SDFs in clinical practice. A study by Robinson and colleagues showed that establishing an electronic referral system between SLPs and pharmacies improved medication administration in dysphagic patients in an acute hospital setting (Robinson et al. 2022). In clinical settings where such systems are not available, patients with dysphagia should ideally undergo a targeted evaluation of SDF swallowability by an SLP—preferably using instrumental methods—especially when new prescriptions are issued. Given the increasing involvement of SLPs in medication‐related decisions, implementing interdisciplinary training programs that cover medication safety and pharmacological considerations for SDF administration could further enhance their competence in this area and improve interprofessional collaboration.

Further results of this study revealed that fruit puree, water and thickened water are the most used accompanying boluses for medication administration. The use of fruit puree aligns with the previously conducted survey on nurses, who stated apple sauce as their preferred bolus. However, the usage of water as the second most favoured bolus contradicts data provided by nurses, who avoid water with SDFs (Trapl‐Grundschober et al. 2025). From a pharmacological perspective, bolus selection may influence drug bioavailability, as specific consistencies can interfere with disintegration, dissolution and absorption processes. These interactions should be considered in clinical decision‐making and further explored in future research (Manrique et al. 2014).

The pathomechanisms and pathological signs that most frequently influence SLPs to recommend a change in SDF administration were pharyngeal phase impairment, cough during medication intake and SDFs remaining in the oral cavity. Pharyngeal swallowing function plays a fundamental role in propelling a bolus and protecting the airway, which could be a possible reason why SLPs prioritise pharyngeal deficits over other pathomechanisms (Speyer et al. 2022). Very little information about the pharyngeal phase of swallowing can be gleaned from the CSE because structures in the pharynx, as well as the pathophysiology behind the aspiration signs (e.g., wet voice, phonation, cough, sensitivity), can only be visualised with the aid of instrumentation (Speyer et al. 2022). Coughing immediately after swallowing is a reliable indicator of either aspiration or penetration (Suiter and Gosa 2019). During a CSE, it may prove challenging to differentiate whether the cough is due to the tablet or the accompanying bolus, which is why instrumental assessment is also preferable when this pathological sign occurs (Wirth and Dziewas 2019; Brady 2015).

No significant differences were found between SLPs who primarily work on stroke units and those who do not. This could be due to the fact that SLPs are typically not assigned to a single ward. Although there was a slightly higher use of screening procedures and FEES in the stroke population, the difference was not statistically significant. In line with this, 70% of the stroke SLPs reported using previously safe‐tested textures, whereas only 49 % of the other group stated this practice. Both results might be related to the increasing usage of FEES on stroke units and the guideline‐based mandatory presence of the SLPs (Dziewas et al. 2024).

Based on these findings, several steps could be considered to improve the assessment and management of SDFs in dysphagic patients. First, standardised protocols should be developed to guide SLPs in evaluating the swallowability of SDFs during both clinical and instrumental assessments. In addition, regulatory frameworks should be adapted to explicitly permit the use of patients’ own medications in swallowing evaluations, as this practice is currently not standardised or legally defined. Second, interdisciplinary guidelines should clarify the role of SLPs in medication‐related decision‐making, ensuring closer integration with pharmacists and physicians. To enhance interprofessional collaboration, structured communication pathways between SLPs, pharmacists and physicians should be established, ensuring a more coordinated approach to medication management in dysphagic patients. Lastly, the implementation of structured training programs on medication management for SLPs could enhance their ability to provide evidence‐based recommendations. The results of this study indicate that SLPs frequently provide recommendations on medication modifications despite receiving little or no formal training in pharmacology, highlighting a clear need for targeted education in this area. These measures could help reduce inconsistencies in clinical practice and improve patient safety.

### 4.1 Limitations

Despite efforts to design the survey to reduce bias, the responses of this survey may not accurately reflect the actual practice patterns due to certain limitations. The pre‐coded response options and inherent limitations of multiple‐choice and Likert‐type responses may not have fully captured the nuanced attitudes and perceptions of all respondents. Due to a convenience sampling method, not all stroke units in German‐speaking countries were included. As SLPs typically work across multiple wards, the available information regarding their working methods on stroke units is inherently limited. Consequently, no significant differences were likely to be found in group comparisons.

Regarding the pathomechanisms influencing SLPs’ decisions for SDF management, it remains unclear whether they are primarily based on CSE or instrumentation, as the questionnaire did not differentiate between these evaluation methods. Lastly, the survey did not investigate to what extent SLPs interact with pharmacists, physicians and nurses regarding the modification of SDFs in clinical practice. This would have demonstrated the interdisciplinary collaboration across different disciplines in clinical settings on this topic.

## 5 Conclusion

This study has identified that a substantial number of SLPs incorporate the evaluation of SDFs into their clinical and instrumental assessments, although standardised tools and guidelines for this area are lacking. SLPs primarily rely on CSE and FEES to assess the swallowability of SDFs. One of the most notable findings is the frequent use of the patient's own medications by SLPs during CSE, contrasting with the administration of placebos during FEES. In addition, this research has also shown that SLPs play a crucial role in recommending the modification of solid medications.

The results of this study may help to enhance the quality of SLP assessment, particularly in relation to patients who require SDF. Future research should focus on establishing standardised protocols and guidelines for the assessment and management of SDFs by SLPs, collaborating with other healthcare professionals, including pharmacists, nurses and physicians, to ensure optimal patient outcomes and medication safety in dysphagic populations. Further empirical investigation is needed to provide a more comprehensive understanding of whether SLPs work differently in the stroke unit setting.

## Ethics Statement

The ethics application for the entire research project was submitted to and reviewed by the Ethics Committee for the Province of Lower Austria. The positive ethics vote was issued on 15th of June 2021 and has already been extended twice (valid until 25.06.2024) (No. GS4‐EK‐4/698‐2021).

## Conflicts of Interest

The authors declare no conflicts of interest.

## Supporting information




**Supporting**: jlcd70073‐sup‐0001‐SuppMat.docx

**Supporting**: jlcd70073‐sup‐0002‐SuppMat.docx

**Supporting**: jlcd70073‐sup‐0003‐SuppMat.docx

## Data Availability

The data that support the findings of this study are not openly available due to reasons of sensitivity and are available from the corresponding author upon reasonable request. Data are located in controlled access data storage at the University of Applied Sciences Wiener Neustadt, Austria: https://www.fhwn.ac.at/en/
