# Supplementary material for: Solid Medication Intake in Hospitalised Patients With Dysphagia: A Challenge for Speech and Language Pathologists?
Source: Int J Lang Commun Disord. 2025 Jul 2;60(4):e70073. doi: 10.1111/1460-6984.70073 (PMC12223472; doi:10.1111/1460-6984.70073)
Supplement: Supplementary file 1 — Supporting: jlcd70073‐sup‐0001‐SuppMat.docx [file JLCD-60-0-s003.docx]

**Title:**

Solid Medication Intake in Hospitalized Patients with Dysphagia: A Challenge for Speech and Language Pathologists?

**Journal:** International Journal of Language & Communication Disorders

**Authors:**

Michaela Trapl-Grundschober^1,2,3^, Lea Schneider^4^, Steffen Schulz^5^, Simon Sollereder^6^, Yvonne Teuschl^7^, Walter Struhal^1,2^, Jürgen Osterbrink^3^

**Affiliation of the corresponding author**

^1^ Karl Landsteiner University of Health Sciences, Dr. Karl-Dorrek-Straße 30, 3500, Krems, Austria

^2^ Division of Neurology, University Hospital Tulln, Alter Ziegelweg 10, 3430, Tulln, Austria

**Corresponding author**

PhDr. Michaela Trapl-Grundschober, MAS, MSc
E-Mail: [michaela.trapl@stud.pmu.ac.at](mailto:michaela.trapl@stud.pmu.ac.at)

**SI 1** Questions of the survey for SLPs_English version

**Assessment of Solid Medication Intake by Speech and Language Pathologists**

Start of the block: Introduction and declaration of consent

**Introduction:** Dear speech and language pathologists! Thank you for taking the time to complete my questionnaire. As part of my second bachelor's thesis in the speech and language therapy degree program at the University of Applied Sciences Wiener Neustadt, I would like to find out how speech and language pathologists in German-speaking countries assess the swallowing of tablets and capsules. The main focus lies on stroke units. However, please also take part in the survey if you work on other wards in a hospital with patients with swallowing disorders. The survey and subsequent evaluation of the responses is completely anonymous; no data sets will be passed on to third parties. No conclusions can be drawn about the person, the hospital or the federal state. By completing this questionnaire, you are making an important contribution to shedding light on this still unexplored topic. Please do not hesitate to contact me if you have any questions!

Best regards,

Lea Schneider

Contact: lea.schneider@fhwn.ac.at

**Declaration of consent:** If you select the answer option "I consent", you consent that data will be collected in anonymized form as part of this questionnaire and used for a Bachelor's thesis. No data records will be passed on to third parties.

- I consent
- I do not consent

Start of the block: Basic Information

Q2.1 Do you work as a speech and language pathologist in a hospital?

- Yes
- No

Q2.2 Which wards do you mainly work on? (multiple choice possible)

- Neurology – Stroke Unit
- Neurology ward
- Intensive care unit
- Internal medicine ward
- ENT ward
- Geriatric ward
- Other(s) ______________________

Q2.3 In which country do you work?

- Austria
- Germany
- Switzerland
- Liechtenstein
- Italy/South Tyrol
- Other country ______________________

Q2.4 How many years of experience do you have with people with dysphagia?

|  | 0 | 5 | 10 | 15 | 20 | 25 | 30 | 35 | 40 | 45 | 50 |
| --- | --- | --- | --- | --- | --- | --- | --- | --- | --- | --- | --- |

| Years | 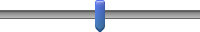 |
| --- | --- |

End of the block: Basic Information

Start of the block: Assessment of swallowing solid oral medication

Q3.1 Does your institution administer medication to patients with suspected dysphagia before the speech and language therapy assessment?

- Yes
- No
- I don’t know

Q3.2 Do you check the swallowing ability of solid oral medication (capsules and tablets) as part of the speech and language therapy assessment for dysphagia by default?

- Yes
- No
- Only in certain exceptional cases (Please describe in the text field if you can think of a specific example). ______________________

End of the block: Assessment of swallowing solid oral medication

Start of the block: Swallowing of solid oral medication is being assessed

Q4.1 Which screening/diagnostic procedure forms the basis for your diagnostics with regard to the suitability of solid oral medications? (multiple choice possible)

- Multi-consistency test (e. g. GUSS)
- Clinical swallowing examination
- Water test (e. g. Daniel’s water swallowing test)
- FEES
- VFSS
- PILL-5
- EAT-10
- Self-designed screening/diagnostic form
- Other ______________________

Q4.2 Have you incorporated the swallowing of capsules and tablets in your self-designed screening/diagnostic form?

- Yes
- No

Q4.3 How do you check the swallowing of tablets or capsules? For the answer(s) that apply to you, please select whether you use instrumental or clinical testing. (multiple choice possible)

|  | Instrumental Assessment (e. g. FEES) | Clinical Assessment (e. g. clinical swallowing examination) |
| --- | --- | --- |
| Placebo tablets |  |  |
| Placebo capsules |  |  |
| Tablets or capsules to be taken by the patient |  |  |
| Other |  |  |

Q4.4 Please select everything that applies to your assessment from the options below. (multiple choice possible)

- I assess different forms of tablets or capsules
- I only assess one tablet or capsule form
- I assess different tablet or capsule sizes
- I only assess one tablet or capsule size
- I only assess the sizes and forms that the patient has to take at the time of my assessment
- I assess the swallowing of crushed placebo tablets or opened placebo capsules
- I assess the swallowing of the patient's own crushed tablets or opened capsules
- Other ______________________

Q4.5 In this context, an accompanying bolus is a substance with which the tablet or capsule is taken together (e. g. water or apple sauce). The accompanying bolus can have different consistencies. Which accompanying bolus consistencies do you most frequently use to check the swallowing of solid oral medication in your assessment? (multiple choice possible)

- IDDSI 0 (thin)
- IDDSI 1 (slightly thick)
- IDDSI 2 (mildly thick)
- IDDSI 3 (moderately thick)
- IDDSI (extremely thick, pureed)
- I only check the consistencies that the patient can swallow safely (previously determined in a general dysphagia assessment)
- Other ______________________

Q4.6 Which types of accompanying bolus do you use most frequently for your assessment? (multiple choice possible)

- Water
- Consistency-modified water (e. g. thickened water or "Nutilis Aqua" from Nutricia)
- Fruit puree (e. g. apple sauce)
- Yoghurt
- Puree
- Baby food (e. g. from the brand "Hipp")
- Swallowing gel (e. g. from the brand "Gloup")
- Other ______________________

End of the block: Swallowing of solid oral medication is being assessed

Start of the block: Parameters

Q5.1 Please indicate what you (help) decide as a speech and language pathologist in your clinic. (multiple choice possible)

- Selection of a different dosage form (e. g. infusions)
- Temporary interruption of oral medication intake
- Administration of medication via nasogastric tube
- Modification of medication (e. g. mortaring or dividing)
- Continuation of oral medication administration without modification
- Other ______________________

Q5.2 Based on your clinical assessment, a patient requires a soft diet (IDDSI 6) and thickened liquids (IDDSI 1). In this case, what do you recommend in terms of medication administration? (multiple choice possible)

- Tablets whole
- Tablets crushed
- Tablets divided
- Capsules whole
- Capsules opened
- Large medication crushed/opened and small medication whole
- Large tablets divided and small tablets whole
- The oral administration of medication is paused
- Other ______________________
- I need more information (Please describe what other information you would need to make a decision) ______________________

Q5.3 Which accompanying bolus do you recommend for the person just described (soft food (IDDSI 6), thickened liquids (IDDSI 1))? (multiple choice possible)

- Thin liquids (IDDSI 0)
- Thickened liquids (IDDSI 1)
- Thickened liquids (IDDSI 2)
- Pureed consistencies (IDDSI 3-4)
- I need more information (Please describe what other information you would need to make a decision) ______________________

Q5.4 Based on your clinical assessment, a patient requires pureed food (IDDSI 3-4) and thickened liquids (IDDSI 2). In this case, what do you recommend in terms of medication administration? (multiple choice possible)

- Tablets whole
- Tablets crushed
- Tablets divided
- Capsules whole
- Capsules opened
- Large medication crushed/opened and small medication whole
- Large tablets divided and small tablets whole
- The oral administration of medication is paused
- Other ______________________
- I need more information (Please describe what other information you would need to make a decision) ______________________

Q5.5 Which accompanying bolus do you recommend for the person just described (pureed food (IDDSI 3-4), thickened liquids (IDDSI 2))? (multiple choice possible)

- Thin liquids (IDDSI 0)
- Thickened liquids (IDDSI 1)
- Thickened liquids (IDDSI 2)
- Pureed consistencies (IDDSI 3-4)
- I need more information (Please describe what other information you would need to make a decision) ______________________

Q5.6 Based on your clinical assessment, a patient should not have any form of food or drink by mouth. In this case, how do you proceed with recommendations regarding the administration of medication? (multiple choice possible)

- Tablets whole
- Tablets crushed
- Tablets divided
- Capsules whole
- Capsules opened
- Large medication crushed/opened and small medication whole
- Large tablets divided and small tablets whole
- The oral administration of medication is paused
- Other ______________________
- I need more information (Please describe what other information you would need to make a decision) ______________________

Q5.7 Which accompanying bolus do you recommend for the person just described (no food or drink by mouth)? (multiple choice possible)

- Thin liquids (IDDSI 0)
- Thickened liquids (IDDSI 1)
- Thickened liquids (IDDSI 2)
- Pureed consistencies (IDDSI 3-4)
- I need more information (Please describe what other information you would need to make a decision) ______________________

Q5.8 On a scale of 0-10, please rate the influence of the following pathomechanisms and symptoms on your decision to adjust/pause oral medication. (0=no influence at all, 10=very great influence)

|  | 0 | 1 | 2 | 3 | 4 | 5 | 6 | 7 | 8 | 9 | 10 |
| --- | --- | --- | --- | --- | --- | --- | --- | --- | --- | --- | --- |

| Disorder of the oral preparatory stage | 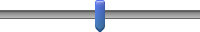 |
| --- | --- |
| Disorder of the oral stage | 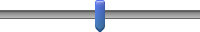 |
| Disorder of the pharyngeal stage | 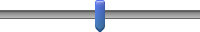 |
| Disorder of the esophageal phase | 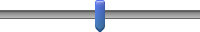 |
| Coughing after/while taking medication | 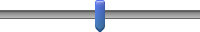 |
| Medication remains in the mouth and cannot be swallowed | 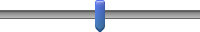 |
| Chewing on medication observable | 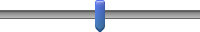 |
| Medication is spit out/rejected | 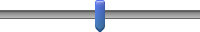 |
| Facial nerve palsy (cranial nerve VII.) | 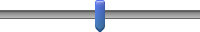 |
| Hypoglossal nerve palsy (cranial nerve XII.) | 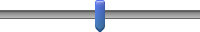 |
| Glossopharyngeal and/or vagus nerve palsy (cranial nerves IX. and X.) | 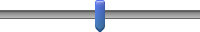 |
| Intraoral sensitivity deficits (cranial nerve V.) | 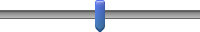 |
| Impairment of more than one cranial nerve relevant to swallowing | 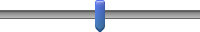 |
| Other | 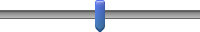 |

End of the block: Parameters

Start of the block: Swallowing of solid oral medication is not being assessed

Q6.1 Why do you not check the swallowing ability of solid oral medication? (multiple choice possible)

- It is not within my area of responsibility in my institution
- In my opinion, it is not within the area of responsibility of speech and language therapy
- There is no standardized assessment for this
- I do not consider this to be relevant
- So far, I do not have considered this problem
- Other __________________________________________________

Q6.2 Based on your dysphagia assessment, do you give recommendations for your patients' medication intake, even if you do not explicitly check this (examples: "mortar medication" or "nil per os except medication")?

- Yes
- No

End of the block: Swallowing of solid oral medication is not being assessed

Start of the block: Personal evaluations

Q7.2 Multi-consistency tests, such as the GUSS (Gugging Swallowing Screen), are tools to assess the swallowing act. Would you find it useful to include checking the swallowing of solid oral medication in these tests?

- Definitely no
- Probably no
- Neither yes nor no
- Probably yes
- Definitely yes

Q7.3 How useful would you find the addition of medication administration recommendations alongside the existing dietary recommendations in these multi-consistency testing procedures?

- Very useless
- Rather useful
- Useful
- Very useful
- Extremely useful

Q7.4 How would you rate your knowledge on diagnosis and management of medication swallowing disorders in people with dysphagia?

- Insufficient
- Below average
- Average
- Good
- Very good

Q7.5 Would you complete further training regarding this topic?

- Definitely no
- Probably no
- Neither yes nor no
- Probably yes
- Definitely yes

Q7.6 What have you always wanted to say about this topic?

________________________________________________________________

End of the block: Personal evaluations
