# Supplementary material for: Solid Medication Intake in Hospitalised Patients With Dysphagia: A Challenge for Speech and Language Pathologists?
Source: Int J Lang Commun Disord. 2025 Jul 2;60(4):e70073. doi: 10.1111/1460-6984.70073 (PMC12223472; doi:10.1111/1460-6984.70073)
Supplement: Supplementary file 2 — Supporting: jlcd70073‐sup‐0002‐SuppMat.docx [file JLCD-60-0-s001.docx]

**Title:**

Solid Medication Intake in Hospitalized Patients with Dysphagia: A Challenge for Speech and Language Pathologists?

**Journal:** International Journal of Language & Communication Disorders

**Authors:**

Michaela Trapl-Grundschober^1,2,3^, Lea Schneider^4^, Steffen Schulz^5^, Simon Sollereder^6^, Yvonne Teuschl^7^, Walter Struhal^1,2^, Jürgen Osterbrink^3^

**Affiliation of the corresponding author**

^1^ Karl Landsteiner University of Health Sciences, Dr. Karl-Dorrek-Straße 30, 3500, Krems, Austria

^2^ Division of Neurology, University Hospital Tulln, Alter Ziegelweg 10, 3430, Tulln, Austria

**Corresponding author**

PhDr. Michaela Trapl-Grundschober, MAS, MSc
E-Mail: [michaela.trapl@stud.pmu.ac.at](mailto:michaela.trapl@stud.pmu.ac.at)

**SI 2** Questions of the survey for SLPs_German version (original)

Erhebung der Schluckfähigkeit fester oraler Medikation durch Logopäd*innen

Beginn des Blocks: Einführung und Einwilligungserklärung

**Einführung:** Sehr geehrte Logopädinnen und Logopäden! Vielen Dank, dass Sie sich die Zeit nehmen, meinen Fragebogen auszufüllen. Im Rahmen meiner zweiten Bachelorarbeit im Studiengang Logopädie an der Fachhochschule Wiener Neustadt möchte ich erheben, wie Logopäd*innen im deutschsprachigen Raum das Schlucken von Tabletten und Kapseln überprüfen. Der Schwerpunkt liegt dabei auf Stroke Units. Bitte nehmen Sie aber auch an der Umfrage teil, wenn Sie auf anderen Stationen in einem Krankenhaus mit schluckgestörten Patient*innen arbeiten. Die Befragung und anschließende Auswertung der Antworten ist komplett anonymisiert, es findet keine Weitergabe von Datensätzen an Dritte statt. Es können weder Rückschlüsse auf die Person, noch auf die Krankenanstalt oder das Bundesland gezogen werden. Durch das Ausfüllen dieses Fragebogens leisten Sie einen wichtigen Beitrag zur Aufklärung dieser noch unerforschten Thematik. Für Fragen stehe ich Ihnen jederzeit zur Verfügung!

Mit freundlichen Grüßen,

Lea Schneider

Kontakt: lea.schneider@fhwn.ac.at</div>

**Einwilligungserklärung:** Wenn Sie die Antwortmöglichkeit „Ich stimme zu“ wählen, sind Sie damit einverstanden, dass im Rahmen dieses Fragebogens Daten in anonymisierter Form erhoben und für eine Bachelorarbeit verwendet werden. Es findet keine Weitergabe von Datensätzen an Dritte statt.

- Ich stimme zu
- Ich stimme nicht zu

Beginn des Blocks: Basisinformationen

Q2.1 Sind Sie als Logopäd*in in einem Klinikum tätig?

- Ja
- Nein

Q2.2 Welche Stationen betreuuen Sie hauptsächlich? (Mehrfachauswahl möglich)

- Neurologie - Stroke Unit
- Neurologie - Normalstation
- Intensivstation
- Interne Station
- HNO-Station
- Geriatrische Station
- Andere Station(en) ______________________

Q2.3 In welchem Land sind Sie tätig?

- Österreich
- Deutschland
- Schweiz
- Liechtenstein
- Italien/Südtirol
- Anderes Land ______________________

Q2.4 Wie viele Jahre Berufserfahrung haben Sie in der Betreuung von Menschen mit Dysphagie?

|  | 0 | 5 | 10 | 15 | 20 | 25 | 30 | 35 | 40 | 45 | 50 |
| --- | --- | --- | --- | --- | --- | --- | --- | --- | --- | --- | --- |

| Jahre | 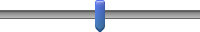 |
| --- | --- |

Ende des Blocks: Basisinformationen

Beginn des Blocks: Diagnostik des Schluckens fester oraler Medication

Q3.1 Werden in Ihrer Institution Medikamente bei Patient*innen mit Verdacht auf Schluckstörung vor der logopädischen Dysphagie-Abklärung verabreicht?

- Ja
- Nein
- Weiß ich nicht

Q3.2 Überprüfen Sie im Rahmen der logopädischen Dysphagie-Abklärung standardmäßig die Schluckfähigkeit fester oraler Medikamente (Kapseln und Tabletten)?

- Ja
- Nein
- Nur in bestimmten Ausnahmefällen (Beschreiben Sie bitte im Textfeld, wenn Ihnen ein konkretes Beispiel einfällt.) ______________________

Ende des Blocks: Diagnostik des Schluckens fester oraler Medikation

Beginn des Blocks: Schlucken fester oraler Medikamente wird überprüft

Q4.1 Welches Screening/diagnostische Verfahren bildet die Grundlage für Ihre Diagnostik in Bezug auf die Eignung fester oraler Medikamente? (Mehrfachauswahl möglich)

- Mehrkonsistenzentestverfahren (z.B. GUSS)
- Klinische Schluckuntersuchung (KSU)
- Wassertest (z.B. Wasserschlucktest nach Daniels)
- FEES
- VFSS
- PILL-5
- EAT-10
- Selbst entworfener Screening-/Diagnostikbogen
- Sonstiges ______________________

Q4.2 Haben Sie in Ihrem selbst entworfenen Screening-/Diagnostikbogen das Überprüfen der Schluckbarkeit von Kapseln und Tabletten schriftlich festgehalten?

- Ja
- Nein

Q4.3 Womit überprüfen Sie das Schlucken von Tabletten oder Kapseln? Bitte wählen Sie für die für Sie zutreffende(n) Antwort(en) aus, ob Sie instrumentell oder klinisch überprüfen. (Mehrfachauswahl möglich)

|  | Instrumentelle Überprüfung (z.B. mittels FEES) | Klinische Überprüfung (z.B. mittels KSU) |
| --- | --- | --- |
| Placebotabletten |  |  |
| Placebokapseln |  |  |
| Vom Patienten/von der Patientin einzuenhmende Tabletten oder Kapseln |  |  |
| Sonstiges |  |  |

Q4.4 Bitte wählen Sie aus den unten angegebenen Auswahlmöglichkeiten alles aus, was auf Ihre Diagnostik zutrifft. (Mehrfachauswahl möglich)

- Ich überprüfe verschiedene Tabletten- oder Kapselformen
- Ich überprüfe nur eine Tabletten- oder Kapselform
- Ich überprüfe verschiedene Tabletten- oder Kapselgrößen
- Ich überprüfe nur eine Tabletten- oder Kapselgröße
- Ich überprüfe nur die Größen und Formen, die der/die Patient*in zum Zeitpunkt meiner Diagnostik einnehmen muss
- Ich überprüfe das Schlucken gemörserter Placebotabletten bzw. geöffneter Placebokapseln
- Ich überprüfe das Schlucken der patient*inneneigenen gemörserten Tabletten bzw. geöffneten Kapseln
- Sonstiges ______________________

Q4.5 Ein Begleitbolus ist eine Substanz, mit der die Tablette oder Kapsel gemeinsam eingenommen wird (z.B. Wasser oder Apfelmus). Dieser kann verschiedene Konsistenzstufen aufweisen. Anhand welcher Begleitbolus-Konsistenzen überprüfen Sie das Schlucken fester oraler Medikation in Ihrer Diagnostik am häufigsten? (Mehrfachauswahl möglich)

- IDDSI 0 (dünnflüssig)
- IDDSI 1 (leicht dickflüssig)
- IDDSI 2 (mäßig dickflüssig)
- IDDSI 3 (stark dickflüssig)
- IDDSI 4 (extrem dickflüssig/breiig, püriert)
- Ich überprüfe nur die Konsistenzen, die der/die Patient*in sicher schlucken kann (vorher in einer allgemeinen Dysphagiediagnostik eruiert)
- Sonstiges ______________________

Q4.6 Welche Arten von Begleitboli verwenden Sie für Ihre Diagnostik am häufigsten? (Mehrfachauswahl möglich)

- Wasser
- Konsistenzmodifiziertes Wasser (z.B. eingedicktes Wasser oder „Nutilis Aqua“ der Firma Nutricia)
- Fruchtmus (z.B. Apfelmus)
- Joghurt
- Brei
- Babynahrung (z.B. der Marke „HiPP“)
- Schluckgel (z.B. der Firma Gloup)
- Sonstiges ______________________

Ende des Blocks: Schlucken fester oraler Medikamente wird überprüft

Beginn des Blocks: Parameter

Q5.1 Bitte geben Sie an, worüber Sie als Logopäd*in in Ihrem Klinikum (mit)entscheiden. (Mehrfachauswahl möglich)

- Auswahl einer anderen Darreichungsform (z.B. Infusionen)
- Vorübergehende Pausierung der oralen Medikamenteneinnahme
- Verabreichung von Medikamenten via Nasogastralsonde
- Modifizierung von Medikamenten (z.B. mörsern oder zerteilen)
- Fortsetzung der oralen Medikamentengabe ohne Modifizierung
- Sonstiges ______________________

Q5.2 Aufgrund Ihrer klinischen Untersuchung benötigt ein/e Patient*in eine weiche Kost (IDDSI 6) und Flüssigkeiten eingedickt (IDDSI 1). Was empfehlen Sie in diesem Fall in Bezug auf die Medikamentenapplikation? (Mehrfachauswahl möglich)

- Tabletten ganz
- Tabletten gemörsert
- Tabletten zerteilt
- Kapseln ganz
- Kapseln geöffnet
- Große Medikamente gemörsert/geöffnet und kleine Medikamente ganz
- Große Tabletten zerteilt und kleine Tabletten ganz
- Die orale Medikamentengabe wird pausiert
- Sonstiges ______________________
- Ich benötige mehr Informationen (Bitte beschreiben Sie, welche Informationen Sie noch zur Entscheidungsfindung brauchen würden.) ______________________

Q5.3 Welchen Begleitbolus (=Substanz, mit der die Tablette oder Kapsel eingenommen wird) empfehlen Sie eben beschriebener Person (weiche Kost (IDDSI 6), Flüssigkeiten eingedickt (IDDSI 1))? (Mehrfachauswahl möglich)

- Flüssigkeiten uneingedickt (IDDSI 0)
- Flüssigkeiten eingedickt (IDDSI 1)
- Flüssigkeiten eingedickt (IDDSI 2)
- Breiige Konsistenzen (IDDSI 3-4)
- Weiche Konsistenzen (IDDSI 6)
- Feste Konsistenzen (IDDSI 7)
- Schluckgels (z.B. der Firma Gloup)
- Sonstiges ______________________
- Ich benötige mehr Informationen (Bitte beschreiben Sie, welche Informationen Sie noch zur Entscheidungsfindung brauchen würden.) ______________________

Q5.4 Aufgrund Ihrer klinischen Untersuchung benötigt ein/e Patient*in eine homogene breiige Kost (IDDSI 3-4) und Flüssigkeiten eingedickt (IDDSI 2). Was empfehlen Sie in diesem Fall in Bezug auf die Medikamentenapplikation? (Mehrfachauswahl möglich)

- Tabletten ganz
- Tabletten gemörsert
- Tabletten zerteilt
- Kapseln ganz
- Kapseln geöffnet
- Große Medikamente gemörsert/geöffnet und kleine Medikamente ganz
- Große Tabletten zerteilt und kleine Tabletten ganz
- Die orale Medikamentengabe wird pausiert
- Sonstiges __________________________________________________
- Ich benötige mehr Informationen (Bitte beschreiben Sie, welche Informationen Sie noch zur Entscheidungsfindung brauchen würden.) __________________________________________________

Q5.5 Welchen Begleitbolus empfehlen Sie eben beschriebener Person (homogene breiige Kost (IDDSI 3-4), Flüssigkeiten eingedickt (IDDSI 2))? (Mehrfachauswahl möglich)

- Flüssigkeiten uneingedickt (IDDSI 0)
- Flüssigkeiten eingedickt (IDDSI 1)
- Flüssigkeiten eingedickt (IDDSI 2)
- Breiige Konsistenzen (IDDSI 3-4)
- Weiche Konsistenzen (IDDSI 6)
- Feste Konsistenzen (IDDSI 7)
- Schluckgels (z.B. der Firma Gloup)
- Sonstiges ______________________
- Ich benötige mehr Informationen (Bitte beschreiben Sie, welche Informationen Sie noch zur Entscheidungsfindung brauchen würden.) ______________________

Q5.6 Aufgrund Ihrer klinischen Untersuchung soll ein/e Patient*in sich oral ernähren. Wie verfahren Sie in diesem Fall mit Empfehlungen in Bezug auf die Medikamentenapplikation? (Mehrfachauswahl möglich)

- Tabletten ganz
- Tabletten gemörsert
- Tabletten zerteilt
- Kapseln ganz
- Kapseln geöffnet
- Große Medikamente gemörsert/geöffnet und kleine Medikamente ganz
- Große Tabletten zerteilt und kleine Tabletten ganz
- Die orale Medikamentengabe wird pausiert
- Sonstiges ______________________
- Ich benötige mehr Informationen (Bitte beschreiben Sie, welche Informationen Sie noch zur Entscheidungsfindung brauchen würden.) ______________________

Q5.7 Welchen Begleitbolus empfehlen Sie eben beschriebener Person (keine orale Ernährung)? (Mehrfachauswahl möglich)

- Flüssigkeiten uneingedickt (IDDSI 0)
- Flüssigkeiten eingedickt (IDDSI 1)
- Flüssigkeiten eingedickt (IDDSI 2)
- Breiige Konsistenzen (IDDSI 3-4)
- Weiche Konsistenzen (IDDSI 6)
- Feste Konsistenzen (IDDSI 7)
- Schluckgels (z.B. der Firma Gloup)
- Sonstiges ______________________
- Ich benötige mehr Informationen (Bitte beschreiben Sie, welche Informationen Sie noch zur Entscheidungsfindung brauchen würden.) ______________________

Q5.8 Bitte schätzen Sie auf einer Skala von 0-10 ein, welchen Einfluss folgende Pathomechanismen und Symptome auf Ihre Entscheidung bezüglich der Anpassung/Pausierung der oralen Medikamentengabe haben. (0=überhaupt keinen Einfluss, 10=sehr großen Einfluss)

|  | 0 | 1 | 2 | 3 | 4 | 5 | 6 | 7 | 8 | 9 | 10 |
| --- | --- | --- | --- | --- | --- | --- | --- | --- | --- | --- | --- |

| Störung der oralen Vorbereitungsphase | 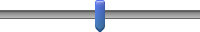 |
| --- | --- |
| Störung der oralen Phase | 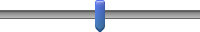 |
| Störung der pharyngealen Phase | 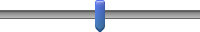 |
| Störung der ösophagealen Phase | 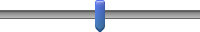 |
| Husten nach/bei der Medikamenteneinnahme | 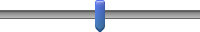 |
| Medikamente verbleiben im Mundraum und können nicht abgeschluckt werden | 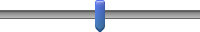 |
| Kauen auf Medikamenten beobachtbar | 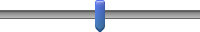 |
| Medikamente werden ausgespuckt/abgelehnt | 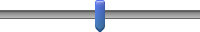 |
| Fazialisparese (VII. HN) | 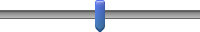 |
| Hypoglossusparese (XII. HN) | 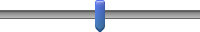 |
| Glossopharyngeus- und/oder Vagusparese (IX. HN und X. HN) | 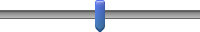 |
| Intraorale Sensibilitätsdefizite (V. HN) | 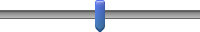 |
| Beeinträchtigung von mehr als einem schluckrelevanten Hirnnerven | 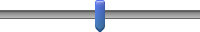 |
| Sonstiges | 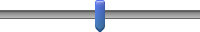 |

Ende des Blocks: Parameter

Beginn des Blocks: Schlucken fester oraler Medikamente wird nicht überprüft

Q6.1 Wieso überprüfen Sie die Schluckfähigkeit fester oraler Medikamente nicht? (Mehrfachauswahl möglich)

- Fällt in meiner Institution nicht in meinen Zuständigkeitsbereich
- Fällt meiner Meinung nach nicht in den Zuständigkeitsbereich der Logopädie
- Es gibt kein standardisiertes Assessment dafür
- Ich erachte dies nicht als relevant
- Ich habe diese Problematik bisher nicht bedacht
- Sonstiges ______________________

Q6.2 Sprechen Sie auf Basis Ihrer Dysphagiediagnostik Empfehlungen für die Medikamenteneinnahme Ihrer Patient*innen aus, auch wenn Sie dies nicht explizit überprüfen (Beispiele: „Medikamente mörsern“ oder „Nil per os außer Medikamente“)?

- Ja
- Nein

Ende des Blocks: Schlucken fester oraler Medikamente wird nicht überprüft

Beginn des Blocks: Persönliche Einschätzungen

Q7.2 Zur Überprüfung des Schluckakts existieren Mehrkonsistenzentests, zu denen beispielsweise der GUSS (Gugging Swallowing Screen) zählt. Würden Sie eine Erweiterung solcher Screeningverfahren auf die Überprüfung des Schluckens fester oraler Medikation sinnvoll finden?

- Definitiv nein
- Wahrscheinlich nein
- Weder ja noch nein
- Wahrscheinlich ja
- Definitiv ja

Q7.3 Wie nützlich wäre für Sie die Ergänzung von Empfehlungen zur Medikamentenapplikation neben den bereits vorhandenen Diätempfehlungen bei diesen Mehrkonsistenzentestverfahren?

- Sehr nutzlos
- Eher nützlich
- Nützlich
- Sehr nützlich
- Extrem nützlich

Q7.4 Wie schätzen Sie Ihr Wissen in Bezug auf Diagnostik und Management von Medikamentenschluckstörungen bei Menschen mit Dysphagie ein?

- Unzureichend
- Unterdurchschnittlich
- Durchschnittlich
- Gut
- Sehr Gut

Q7.5 Würden Sie Fortbildungen in Bezug auf diese Thematik besuchen?

- Definitiv nein
- Wahrscheinlich nein
- Weder ja noch nein
- Wahrscheinlich ja
- Definitiv ja

Q7.6 Was wollten Sie zu diesem Thema schon immer anmerken?

________________________________________________________________

Ende des Blocks: Persönliche Einschätzungen
