# Supplementary material for: Solid Medication Intake in Hospitalised Patients With Dysphagia: A Challenge for Speech and Language Pathologists?
Source: Int J Lang Commun Disord. 2025 Jul 2;60(4):e70073. doi: 10.1111/1460-6984.70073 (PMC12223472; doi:10.1111/1460-6984.70073)
Supplement: Supplementary file 3 — Supporting: jlcd70073‐sup‐0003‐SuppMat.docx [file JLCD-60-0-s002.docx]

**Title:**

Solid Medication Intake in Hospitalized Patients with Dysphagia: A Challenge for Speech and Language Pathologists?

**Journal:** International Journal of Language & Communication Disorders

**Authors:**

Michaela Trapl-Grundschober^1,2,3^, Lea Schneider^4^, Steffen Schulz^5^, Simon Sollereder^6^, Yvonne Teuschl^7^, Walter Struhal^1,2^, Jürgen Osterbrink^3^

**Affiliation of the corresponding author**

^1^ Karl Landsteiner University of Health Sciences, Dr. Karl-Dorrek-Straße 30, 3500, Krems, Austria

^2^ Division of Neurology, University Hospital Tulln, Alter Ziegelweg 10, 3430, Tulln, Austria

**Corresponding author**

PhDr. Michaela Trapl-Grundschober, MAS, MSc
E-Mail: [michaela.trapl@stud.pmu.ac.at](mailto:michaela.trapl@stud.pmu.ac.at)

**SI 3** Reasons for exceptional cases to assess solid dosage forms

Question Q3.2: Do you routinely assess the swallowing ability of solid oral medication (capsules and tablets) as part of the clinical swallowing assessment?

37 participants indicated to do this only in exceptional cases. 28 stated the following reasons for the exceptions:

English translation

1. At the request of the nurse
2. Only on stroke units
3. In case of anomalies in the medical history
4. In the event of difficulties or a change in medication
5. In the case of residual symptoms in patients who have recently started oral diet
6. Placobo in FEES, or medications after medical referral on stroke units
7. Frequently but not in every patient
8. Frequently during FEES
9. During FEES
10. Standardized during FEES, CSE: in individual cases only
11. During FEES
12. Yes: FEES; No: CSE
13. M.Parkinson in on-off phases
14. Only on request of the ward
15. Only in patients who are allowed to eat and drink orally
16. Only if other consistencies are conspicuous
17. Only if the patient has to take his/her medicationat the time of the CSE
18. At the request of the nurse
19. The evaluation is conducted during mealtime assessments
20. In case of anomalies in the medical history and standardized during FEES
21. In the event of difficulties observed by nurses or patients themselves
22. Only if other consistencies are conspicuous (puree, soft, solid)
23. In the event of difficulties observed by nurses
24. In case of removing the NGT, or the CSE is good enough, or if the patient has to take his/her medication at the time of the CSE
25. at the request of the nurse or or if the patient has to take his/her medication at the time of the CSE
26. If medications are not allowed to be crushed
27. In the event of difficulties observed by nurses or patients themselves
28. Only if the patient has to take his/her medication at the time of the CSE

German:

1. Auf Bitte der Pflege
2. Auf der Stroke unit schon
3. Bei Auffälligkeiten in der Anamnese
4. Bei auftretenden Schwierigkeiten oder Umstellung von Medikamenten
5. Bei Restsymptomatik, bei frisch oral kostaufgenauten Pat.
6. Entweder Placebo in der Fees oder Medikamente nach Anweisung auf der Stroke Unit
7. häufig aber nicht bei jedem Patienten
8. häufig im Rahmen der FEES
9. im Rahmen der FEES-Untersuchung
10. In der FEES standardisiert, in der KSU nur im Einzelfall
11. Innerhalb der FEES
12. Ja, bei FEES Untersuchungen; nein bei klinischer Schluckuntersuchung
13. M. Parkinson in off/on- Phasen
14. Nur auf Nachfrage der Stationen
15. Nur bei PatientInnen, die oralisiert werden können und über ab Koststufe 2.
16. Nur wenn andere Konsistenz auffällig sind
17. Nur wenn der Pat. in dem Moment der Schluckbeurteilung auch Medikamente nehmen sollte (wenn es zeitlich zusammenpasst)
18. Nur wenn von Pflege gefragt
19. Überprüfung verläuft i.R. von Dysphagie Verlaufskontrollen bei den Mahlzeiten
20. Wenn anamnestisch das Schlucken von Tabletten als schwierig angegeben wird und auch standardmäßig in der FEES, insofern das Schlucken zuvor auffällig war
21. Wenn der Pflege auffällt dass es hierbei Probleme gibt oder wenn der Patient Beschwerden äußert
22. Wenn es Auffälligkeiten bei den konsistenzen püriert, weich, fest gibt.
23. Wenn es bereits Auffälligkeiten seitens der Pflege gibt.
24. wenn es um Entfernung von NGS geht od. wenn Pat. bei KSU "gut" genug sind um dies zu testen u. gerade Medika verabreicht werden sollten
25. Wenn gerade zufällig welche da sind oder die Pflege Probleme beschreibt
26. wenn Medikamente nicht zermörsert werden dürfen bzw. in fester Form eingenommen werden müssen
27. Wenn Patient*innen oder die Pflege Schwierigeiten beim Medikamente schlucken angeben
28. Wenn Tabletten zum Zeitpunkt der Abklärung ein zu nehmen sind, werden sie standardmäßig mitgetestet. Es werden aber keine Placebotabletten nur zu Testzwecken verabreicht.
